# Targeted nanotherapeutics for the treatment of *Helicobacter pylori* infection

**DOI:** 10.1186/s12929-024-01068-9

**Published:** 2024-08-11

**Authors:** Rute Chitas, Diana R. Fonseca, Paula Parreira, M. Cristina L. Martins

**Affiliations:** 1 i3S - Instituto de Investigação e Inovação em Saúde, Universidade do Porto, Porto, Portugal; 2 INEB - Instituto de Engenharia Biomédica, Universidade do Porto, Porto, Portugal; 3 ICBAS - Instituto de Ciências Biomédicas Abel Salazar, Universidade do Porto, Porto, Portugal; 4 FEUP - Faculdade de Engenharia, Departamento de Engenharia Metalúrgica e de Materiais, Universidade do Porto, Porto, Portugal

**Keywords:** *Helicobacter pylori*, Bioengineered strategies, Nanotherapeutics, Nanoparticles, Microparticles, Targeted therapies

## Abstract

*Helicobacter pylori* infection is involved in gastric diseases such as peptic ulcer and adenocarcinoma. Approved antibiotherapies still fail in 10 to 40% of the infected patients and, in this scenario, targeted nanotherapeutics emerged as powerful allies for *H. pylori* eradication. Nano/microparticles conjugated with *H. pylori* binding molecules were developed to eliminate *H. pylori* by either (i) blocking essential mechanisms of infection, such as adhesion to gastric mucosa or (ii) binding and killing *H. pylori* through the release of drugs within the bacteria or at the site of infection. Glycan antigens (as Lewis B and sialyl-Lewis X), pectins, lectins, phosphatidylethanolamine and epithelial cell membranes were conjugated with nano/microparticles to successfully block *H. pylori* adhesion. Urea-coated nanoparticles were used to improve drug delivery inside bacteria through *H. pylori* UreI channel. Moreover, nanoparticles coated with antibodies against *H. pylori* and loaded with sono/photosensitizers, were promising for their application as targeted sono/photodynamic therapies. Further, non-specific *H. pylori* nano/microparticles, but only active in the acidic gastric environment, coated with binders to bacterial membrane, extracellular polymeric substances or to high temperature requirement A protease, were evaluated. In this review, an overview of the existing nanotherapeutics targeting *H. pylori* will be given and their rational, potential to counteract infection, as well as level of development will be presented and discussed.

## Introduction

*Helicobacter pylori* is recognized as a gastric pathogen associated to the development of several gastric disorders, including gastric cancer [1–3]. This bacterium is ubiquitously distributed, being estimated that it infects more than half of the world population [4, 5]. This high colonization rate is associated with several features that enable *H. pylori* to thrive in the harsh gastric environment [6], namely: (i) urease—an enzyme that hydrolyzes urea in ammonia and carbon dioxide, neutralizing the gastric acid in the bacteria vicinity and reducing mucins viscosity, which facilitates *H. pylori* mobility through the mucus layer [6, 7]; (ii) spiral shape and flagella—to cross the mucus layer and reach the neutral gastric epithelium [8, 9]; (iii) adhesins—that specifically adhere to glycans expressed on the mucus layer and on the gastric epithelium, granting protection against stomach displacement (e.g. peristaltic movements) [10, 11]; iv) morphological plasticity—from spiral to coccoid-shape as a defense against adverse conditions and v) biofilms—that shield the bacterium from antimicrobial agents, increasing treatment failure and infection recrudescence [12, 13]. As 90% of gastric cancers are linked to *H. pylori* infection, in some countries eradication is now advised for all infected patients independently of symptomatology [14]. The commonly prescribed therapies are based on a combination of broad-spectrum antibiotics and proton pump inhibitors [7, 14]. However, their efficacy dramatically decreased over the years, with eradication rates reaching as low as 70%, a value well below the 90% defined as acceptable by the Maastricht Consensus Report [14, 15]. This failure has been mainly associated to the development of antibiotic resistance allied to the low patient compliance to the complex therapeutic scheme but also to the drugs low stability and bioavailability in the gastric environment [7, 16]. Additionally, since these treatments are based on the use of broad-spectrum antibiotics, they often alter the gut microbiota triggering dysbiosis, which further negatively impacts human health [17, 18]. Altogether, this prompt the World Health Organization (WHO) to include *H. pylori* on the list of the 12 most critical antibiotic-resistant bacteria that must be prioritized for developing new antibiotics therapeutics [19].

On this topic, different antibiotic-free therapeutic strategies have been explored, namely focused on probiotics, antimicrobial peptides and phytocompounds [1, 7, 20–22]. Additionally, vaccines have been explored to prevent *H. pylori* infection [20, 23]. However, although all these strategies had potential against *H. pylori,* their efficacy was low when translated to in vivo or in clinical trials [21, 24]. Similar to what was reported for antibiotics, these strategies also had low stability (e.g. oxidation, proteolysis) in the harsh gastric environment [15, 21, 22]. To overcome their bioavailability issue, the use of nano/microsystems (nanoparticles ( < 1000 nm; NP) and microparticles (≥ 1000 nm; MP)) has been explored [21, 25, 26]. These systems can be classified as lipidic, polymeric or metallic, according to the biomaterial chosen [25, 27]. The most common nano/microparticles classes, the biomaterials used for their production and their properties in gastric settings and against *H. pylori* are described in Table 1.

**Table 1:** Common nano/microparticles classes, examples of their components and main properties for gastric settings

Class	Type	Biomaterial	Properties	Ref
Polymeric	Nanoparticles Microparticles	Chitosan	Mucoadhesive Antimicrobial	[28, 29]
		Polylactic-co-glycolic acid (PLGA)	Biocompatible Optimal control of drug release in vivo Adequate for several active ingredients	[30]
		Gliadin	Mucoadhesive	[31]
Lipidic	Liposomes Nanoparticles	Lipids (e.g. phosphatidylethanolamine)	Biocompatible Adequate for lipophilic and hydrophilic drugs, Easily modified to contain mucoadhesive properties	[32]
Metallic	Nanoparticles	Gold	Antimicrobial Antitumor Mucoadhesive Used in theragnostic applications	[33, 34]
Others	Nanomicelles	Several (e.g. hyaluronic acid, chitosan)	Biocompatible pH‐responsive Mucoadhesive Adequate for hydrophobic drugs	[35, 36]
	Nanocomposites	Several (e.g. polymeric or metallic nanoparticles)	Multiphase nanomaterial Combine different nanoparticles and their properties Accumulates the advantages of all the nanoparticles involved	[37]

## *H. pylori* targeted nano/microsystems

**Fig. 1:**
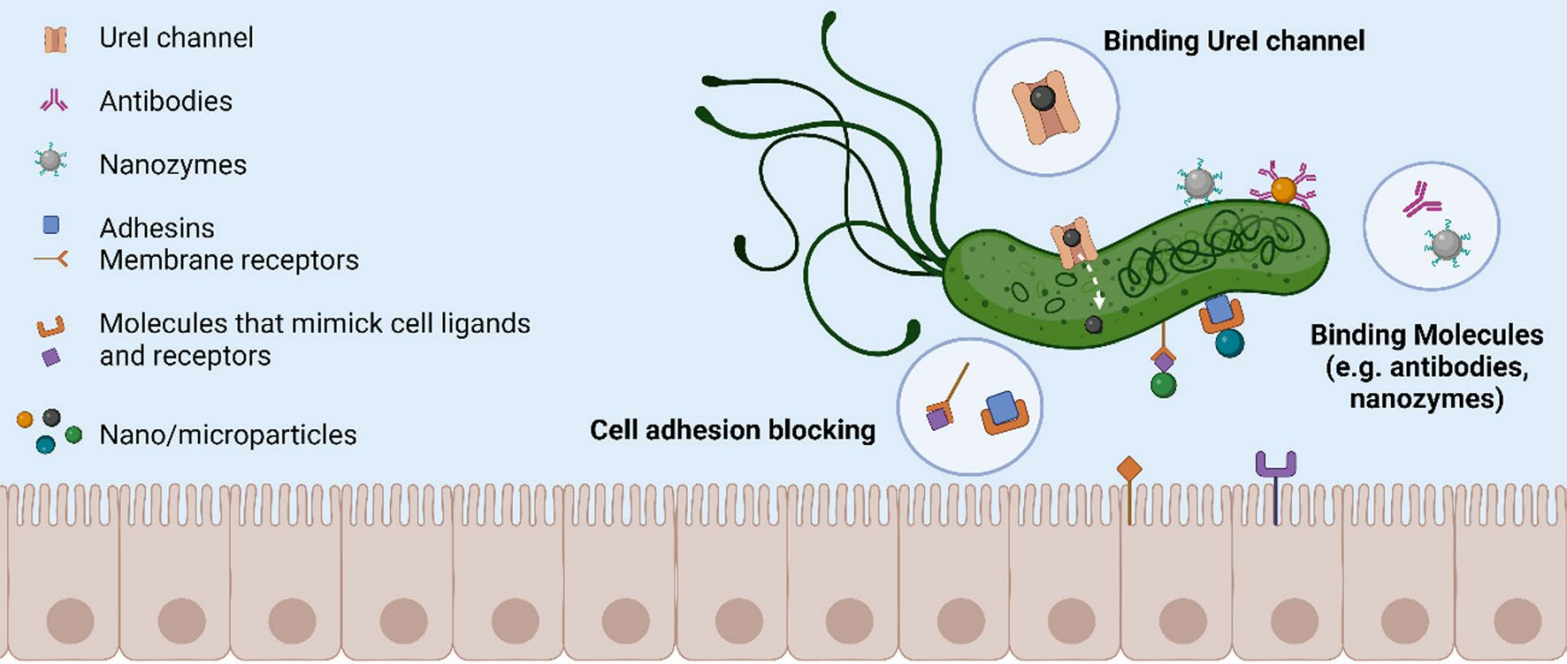
Different *H. pylori* targeting nano/microsystems approaches. (Figure not to scale, created with BioRender.com)

The above-mentioned NP/MP can be conjugated with *H. pylori* binding molecules to promote targeted therapies [26] based on: (i) blocking *H. pylori* adhesion to host gastric cells; (ii) releasing drugs inside bacteria after specific binding to the UreI channel or (iii) binding to the *H. pylori* membrane for a localized drug delivery. A schematic representation of how the target molecules interact with *H. pylori* is shown in Fig. 1.

This review explores the existing nano/microsystems specifically designed to target and eradicate *H. pylori* without affecting the gut microbiota. They were organized according to their ability to: (i) block *H. pylori* adhesion; (ii) bind *H. pylori* UreI channel and (iii) bind *H. pylori* membrane. Within each section they were divided by the *H. pylori* binding molecules used for its targeting (Table 2).

**Table 2:** Identification of the main *H. pylori* binding molecules used in nano/microsystems for *H. pylori* targeting

Binding molecules	Particle	Antibiotics	Exp. phase	Eradication ≥ 90%	Ref
Le^b^/sLe^x^	Chitosan MP	No	In vivo (Le^b^ transgenic C57BL/6 mice)	No	[40]
Pectin	Liposomes	AMX	In vitro	ND	[47]
	Lipid polymer NP	AMX	In vitro	No	[49]
Lectin	Giadin NP	No	In vitro	Yes	[55]
	Giadin NP	CLR, AMX & OMZ	In vivo (Swiss albino mice)	No	[58]
	PLGA NP	CLR	In vitro	*	[59]
Fucose	Chitosan/heparin NP	AMX	In vivo (C57BL/6J mice)	No	[52]
	Chitosan/gelatin NP	No	In vivo (C57BL/6J mice)	No	[62]
	CMC & Au NP	No	In vivo (unspecified mice strain)	*	[33]
	Chitosan NP	No	In vivo (C57BL/6 mice)	No	[63]
	Metformin & LA NP	No	In vivo (C57BL/6 mice)	Yes	[65]
Mannose	Chitosan NP	No	In vitro	*	[54]
PE and fucose	Liposomes	AMX & MTZ	In vitro	*	[76]
PE	Double liposomes	AMX	In vitro	No	[77]
	PVA NP (nanolipobeads)	AMX	In vivo (Albino rats)	Yes	[79]
	Lipidic NP	AMX	In vitro	Yes	[80]
Cell membranes	PLGA NP	CLR	In vivo (C57BL/6 mice)	Yes	[81]
Urea	Nanomicelles	CLR	In vivo (Wistar rats)	Yes	[88]
	Nanomicelles	CLR	In vivo (unspecified mice strain)	ND	[93]
	Chitosan NP	AMX	In vitro	No	[89]
	PLGA NP	AMX	In vivo (BALB/c mice)	No	[30]
	Chitosan+CDs NP	AMX	In vivo (C57BL/6 mice)	No	[95]
Antibodies	Liposomes	No	In vivo (BALB/c mice)	No	[98]
	Au NP	No	In vivo (BALB/c mice)	Yes	[100]
Dextran sulfate	NE	No	In vitro	*	[110]
Boronic acid	Graphene Nanozymes	No	In vivo (BALB/c mice)	Yes	[112]
	MS-Chitosan-Au Nanozymes	No	In vivo	*	[113]
Montmorillonite	Clay NP	MTZ	In vivo (BALB/c mice)	*	[119]
JO146	PLGA NP	No	In vitro	*	[122]
Apigenin	Eudragit RS 100 microsponge	No	In vitro	*	[121]

Discrimination of the use of antibiotics, experimental phase, and bactericidal efficacy

*PE* phosphatidylethanolamine, *NP* nanoparticles, *MP* microparticles, *PLGA* polylactic-co-glycolic acid, *PVA* poly vinyl alcohol, *EC* ethlylcellulose, *CMC* carboxymethyl chitosan, *LA* linoleic acid, *CDs* carbon dots, *Au* gold, *NE* nanoemulsions, *MS* mesoporous silica, *CLR* clarithromycin, *AMX* amoxicillin, *OMZ* omeprazole, *MTZ* metronidazole, *ND* no data

^*^Qualitative assessment

### Block *H. pylori* adhesion

The ability to adhere to the mucus layer and gastric epithelial cells is key for a successful infection. *H. pylori* adhesion is mediated by a variety of adhesins and most of them are outer membrane proteins (OMP) that can recognize specific glycans expressed in the host mucus layer and cellular membrane [10]. OMP binding molecules were used for coating micro- and nanoparticles to bind *H. pylori* OMP and inhibit the adhesion step by competing with the glycans present in gastric epithelium. In addition, these systems can transport antibiotics or other antimicrobial compounds to be released at the bacteria vicinity, improving the treatment efficiency. Some *H. pylori* binding blockers that have been used as nano/microparticles coatings are: (i) Lewis b (Le^b^) and sialyl-Lewis x (sLe^x^) antigens; (ii) Pectins; (iii) Lectins; (iv) Phosphatidylethanolamine and (v) Epithelial cell membranes.

#### Lewis b (Le^b^) and sialyl-Lewis x (sLe^x^) antigens

**Fig. 2:**
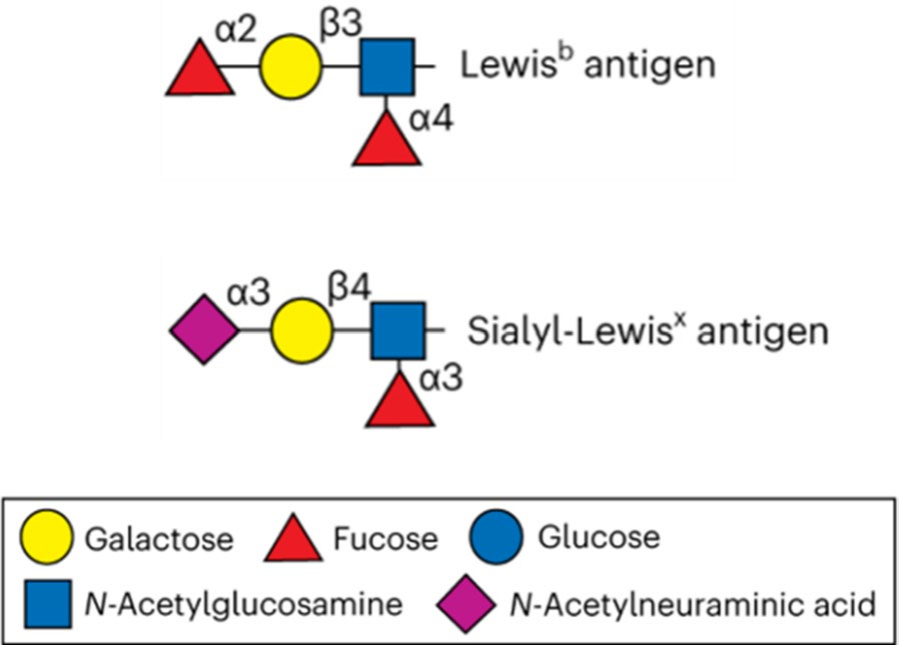
Schematic structure of Lewis b (Le^b^) and sialyl-Lewis x (sLe^x^) antigens expressed in human gastric mucosa. Adapted with permission from [39]

Le^b^ and sLe^x^ antigens (Fig. 2) are carbohydrates (glycans) that are specifically recognized by *H. pylori* adhesins mediating the interaction between the bacterium and the host cells. Among them, the blood group antigen binding adhesin (BabA) that recognizes Le^b^ and the sialic acid-binding adhesin (SabA) which recognizes sLe^x^, are the most prominent [38].

**Fig. 3:**
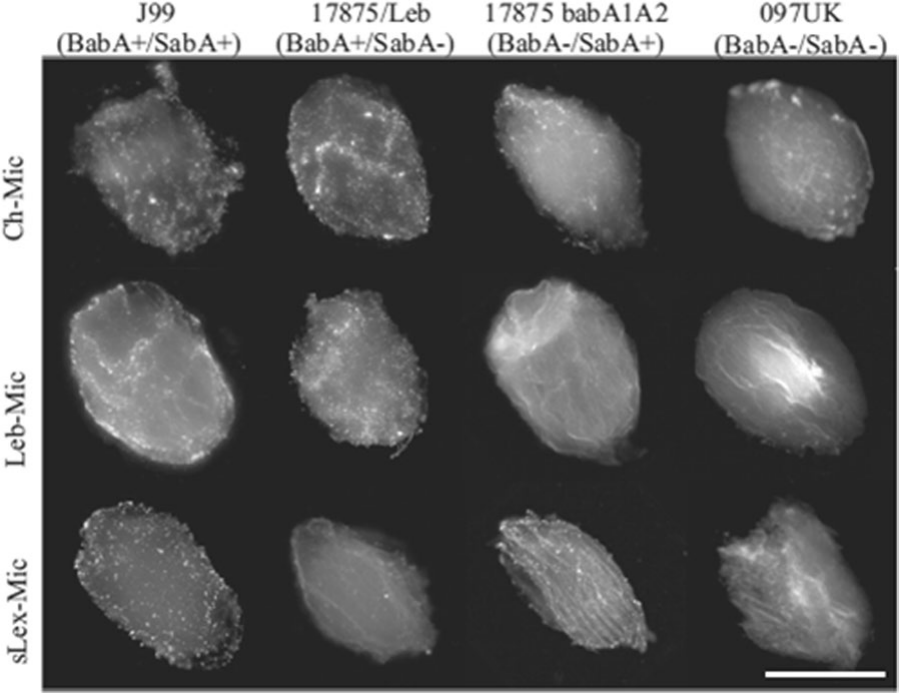
Fluorescence microscopy images of fluorescein isothiocyanate (FITC)-labeled *H. pylori* strains with distinct adhesins profile adhered to different glycans-ChMP (visible due to auto-fluorescence) at an excitation wavelength of 488 nm. Bacteria are represented as brighter dots on the image. *H. pylori* strains were able to bind nonspecifically to ChMP (control; without glycans) independently of the adhesin profile. Bacteria bound specifically to glycans-ChMP with a compatible glycan/adhesin profile, with BabA^+^ *H. pylori* strains (J99 and 17875/Leb) binding to Le^b^-ChMP and SabA^+^ *H. pylori* strains (J99 and 17875 babA1A2) binding to sLe^X^-ChMP. Scale bar = 100 µm. Adapted with permission from [40]

To specifically remove *H. pylori* from the gastric tract, Le^b^ and sLe^x^ glycans were grafted onto chitosan (a mucoadhesive polysaccharide, FDA approved for oral administration and widely explored in drug delivery systems for gastric applications) microspheres (ChMP) [40, 41]. The targeting potential was demonstrated by using *H. pylori* strains with different adhesins profile: *H. pylori* J99 (BabA^+^/SabA^+^), *H. pylori* 17875/Leb (BabA^+^/SabA^−^), *H. pylori* 17875babA1A2 (BabA^−^/SabA^+^) and *H. pylori* 097UK (BabA^−^/SabA^−^). The specific binding between microspheres and bacteria with compatible glycans/adhesins was confirmed, with BabA^+^ strains binding to Le^b^-ChMP and SabA^+^ strains binding preferentially to sLe^x^-ChMP (Fig. 3). Le^b^-ChMP were also tested in 2D (human gastric tissue sections) and 3D (Le^b^ transgenic C57BL/6 mice stomachs) models infected with *H. pylori* 17875/Leb (BabA^+^/SabA^−^), as both models express Le^b^ glycans (to compete for *H. pylori* adhesion). In human tissues, Le^b^-ChMP removed 43% of *H. pylori* 17875/Leb previously adhered to the mucosa and prevented bacterial adhesion in 35%, whereas in control samples (ChMP) adhesion was only reduced in 25%. In the 3D ex vivo mice model, Le^b^-ChMP performance was enhanced, removing 65% of adhered *H. pylori* 17875/Leb and preventing 78% of adhesion [40]. Thus, the grafting of these glycans successfully targeted *H. pylori* accordingly to their adhesins profile and inhibited bacterial adhesion to the gastric mucosa. However, not all *H. pylori* strains express these adhesins and their expression is not constitutive (e.g. SabA expression is triggered by inflammation) what can hamper the efficacy of this strategy for universal eradication [10].

#### Pectins

**Fig. 4:**
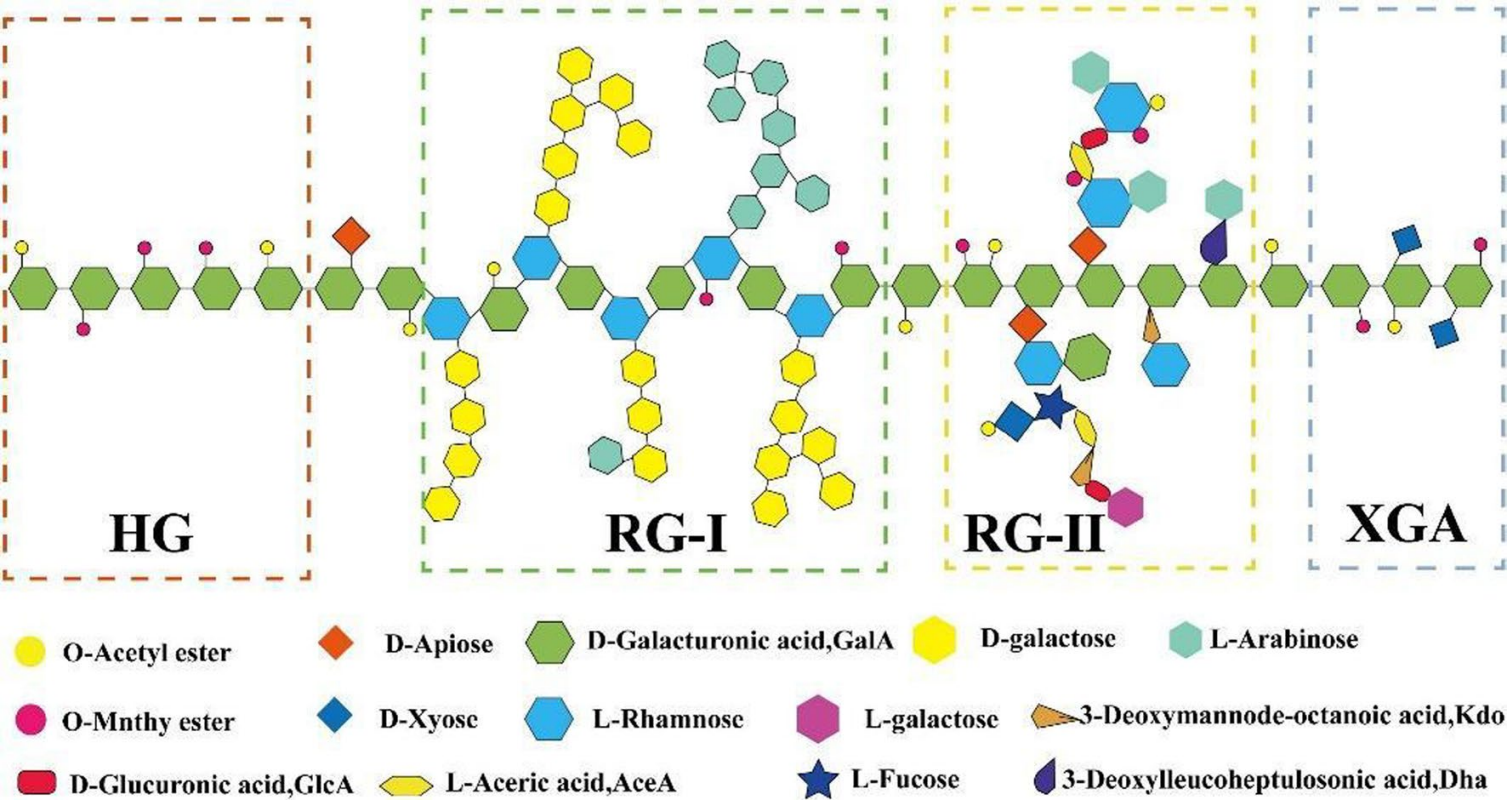
Schematic pectin structure. Pectins are constituted by different polysaccharide motifs: homogalacturonan (HG), rhamnogalacturonan I (RG-I), rhamnogalacturonan II (RG-II) and xylogalacturonan (XGA). Adapted with permission from [43]

Pectins are polysaccharides composed by different polysaccharide motifs (Fig. 4) that are commonly found in fruits and vegetables [42].

Due to their structure rich in monosaccharides, similar to those found in the Lewis antigens of gastric cells, pectin can bind to *H. pylori* BabA adhesin [44, 45]. In addition, due to its highly hydrophilic polysaccharide composition, pectin is also known by its anti-adhesive properties [46]. Thus, nanosystems with pectin are envisioned to penetrate the mucus layer and bind to *H. pylori*, competing with its adhesion to gastric cells [45].

**Fig. 5:**
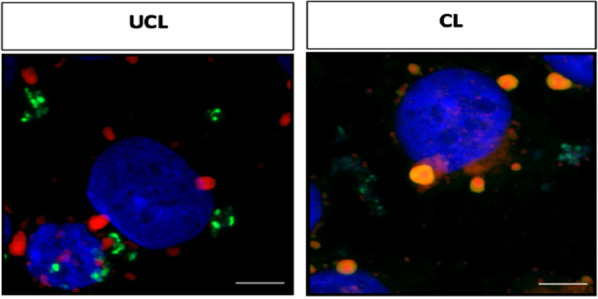
Representative confocal laser scanning microscopy images of human gastric carcinoma epithelial cell line (AGS) cells incubated with *H. pylori* and liposomes (UCL, uncoated liposomes; CL, coated liposomes). Cell nuclei are stained with 4′,6-diamidino-2-phenylindole (DAPI, blue), bacteria with fluorescein isothiocyanate (FITC, green), and liposomes with 1,1-dioctadecyl-3,3,3,3- tetramethylindodicarbocyanin (DiD, red). Orange: *H. pylori* co-localized with liposomes. Scale bar 5 μm. Magnification: 63x. Adapted with permission from [47]

The capacity of pectin to target *H. pylori* was demonstrated by Gottesmann et al*.* with pectin coated liposomes (CL) and *H. pylori* J99 strain [47]. The specific binding of CL to *H. pylori* was confirmed by confocal microscopy using labeled CL and labeled liposomes without pectin coating (UCL) (Fig. 5).

After incubation of human gastric carcinoma epithelial cell line (AGS) with *H. pylori* and labeled liposomes (CL and UCL), only the pectin coated liposomes (CL) interacted with the bacterium, confirming the targeting potential of pectin. However, it was also demonstrated that CL did not prevent *H. pylori* adhesion to cells. Formulations with amoxicillin (AMX) with or without pectin coating (CL-AMX and UCL-AMX, respectively), showed similar concentration dependent antimicrobial effect, whereas the control liposomes (without AMX) did not display antimicrobial activity, as expected [47]. Therefore, despite no advantages being observed in terms of antibiotic delivery or in cell binding inhibition, it was established that the presence of pectin improved liposome interaction with *H. pylori*, which can be further explored for the design of other *H. pylori* targeted strategies.

Pectin sulfate (PECS) was also evaluated for its capacity to mimic specific oligosaccharide epitopes of mucins and glycosaminoglycans found on the host cells that are rich in sulfate groups and are recognized by *H. pylori* OMP [48]. After confirming that soluble PECS bound to *H. pylori* and inhibited its adhesion to AGS cells [48], these were further explored in a lipid polymer nanocarrier (LPN) system and tested against *H. pylori* biofilms (more resistant to antibiotics and other antimicrobial compounds than planktonic bacteria) [49]. This LPN system (PECS-RHL-LPN) encompassed a rhamnolipid (RHL) known to disrupt the biofilms extracellular polymeric substances (EPS), while PECS were included to inhibit bacterial adhesion to gastric cells and PECS-RHL-LPN were able to protect AGS cells from *H. pylori* infection. Then, to further add antimicrobial potential, AMX was loaded in this LPN system (AMX-PECS-RHL-LPN) [49]. When tested in biofilms, AMX-PECS-RHL-LPN killed 70–80% of *H. pylori*. This bactericidal activity was significantly lower in LPN without RHL (≈60%) and in the controls of soluble PECS+AMX (≈20%) and AMX in solution (≈10%) [49]. Additionally, the minimum inhibitory concentration (MIC) was reduced from 125 µg/mL (AMX in solution) to 15.6 µg/mL (AMX-PECS-RHL-LPN), highlighting that AMX-PECS-RHL-LPN improved AMX delivery on biofilms [49]. Although this strategy showed promising results against *H. pylori* biofilms, in vivo studies were not conducted to date.

#### Lectins

Lectins are saccharide-binding glycoproteins that are expressed in many organisms from plants to animal cells, usually involved in cell adhesion and protein synthesis regulation [50]. These proteins are generally isolated from vegetal sources and can be classified according to the saccharide for which they have affinity [51]. Some of the most common lectins bind to fucose (e.g. *Ulex europaeus* agglutinin) and mannose (e.g. Concanavalin A). *H. pylori* membrane contains lectins that bind selectively to fucose and mannose residues present at the gastric mucosa. For example, fucose targets *H. pylori* BabA, blocking *H. pylori* adhesion to the fucosylated Le^b^ antigen in the host gastric mucosa [52–54]. *H. pylori* also has carbohydrate receptors with lectins affinity on its membrane and thus, two strategies were developed: (a) lectin coated NP targeting *H. pylori* membrane carbohydrate receptors and (b) fucose or mannose coated NP targeting *H. pylori* membrane lectins.

##### Lectins—NP

**Fig. 6:**
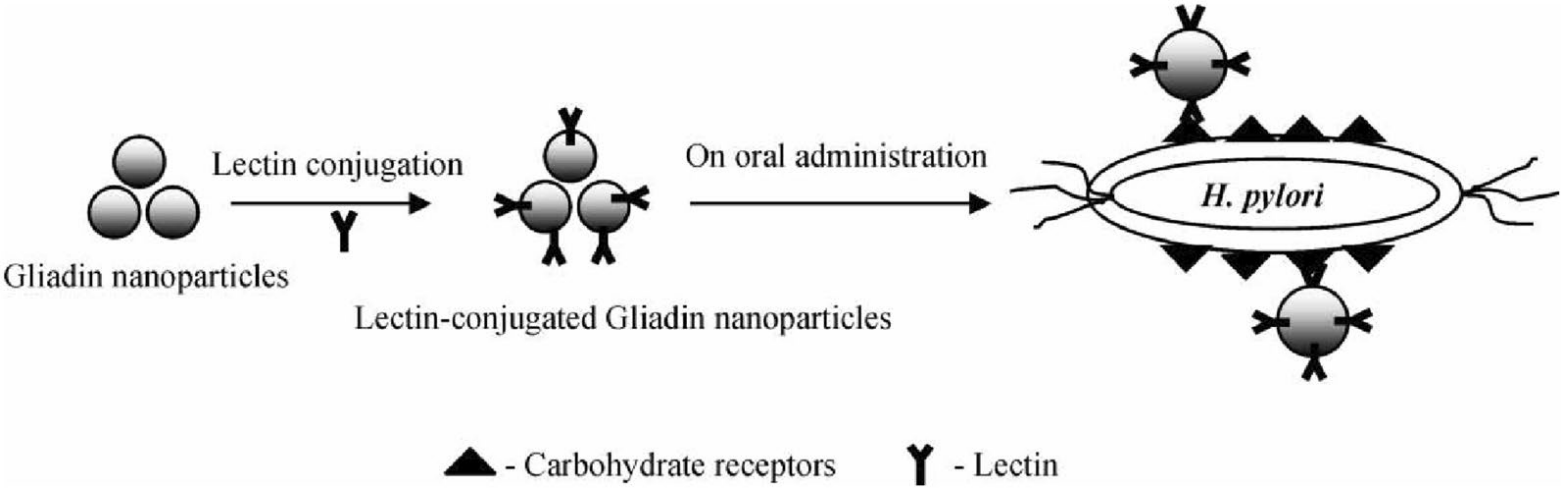
Recognition of lectin-conjugated formulations by carbohydrate receptors on *H. pylori* surface. Used with permission from [55]

Two lectins were surface conjugated (covalent binding) on gliadin (a mucoadhesive glycoprotein usually found in gluten) nanoparticles (G NP): mannose specific Concanavalin A (Con A) and the fucose specific *Ulex europaeus* agglutinin I (UEA-I) (Fig. 6) [31].

To confer antibacterial properties, acetohydroxamic acid (AHA), a urease inhibitor, was added to the formulation. When tested against *H. pylori* NCTC 11637 strain*,* both UEA-G and ConA-G NP inhibited more than 90% of bacterial growth after 12 h, a superior performance when compared to equal amounts of G NP (48%) or AHA in solution (75%) [55].

The potential of this system was further explored using ConA-G NP loaded with drugs commonly used in *H. pylori* triple therapy, namely clarithromycin (CLR) and AMX and the proton pump inhibitor omeprazole (OMZ) [56–58]. When tested against *H. pylori* (in vitro) ConA-G NP loaded with those three drugs achieved 95% of bacterial growth inhibition in contrast with ConA-G NP loaded with just one of the drugs, which achieved 67% (CLR), 58% (AMX) and 32% (OMZ) of growth inhibition. In vivo (Swiss albino mice) efficacy assays also showed that the triple therapy loaded onto ConA-G NP yielded better performance with 83% of eradication rate versus 67% for NP without ConA and 33% for triple therapy in solution [58]. ConA-conjugated poly (lactic-co-glycolic acid) (PLGA) nanoparticles loaded with CLR and AHA were also designed. When tested against *H. pylori* 1101 strain, these NP had a lower (1.1 µg/mL) MIC than CLR (8.9 µg/mL), AHA (75 µg/mL) and CLR + AHA (7.3 µg/mL), validating these NP as a *H. pylori* specific treatment. However, to date, no follow-up was done in vivo [59].

##### Lectin-binding NP (Fucose and Mannose-NP)

**Fig. 7:**
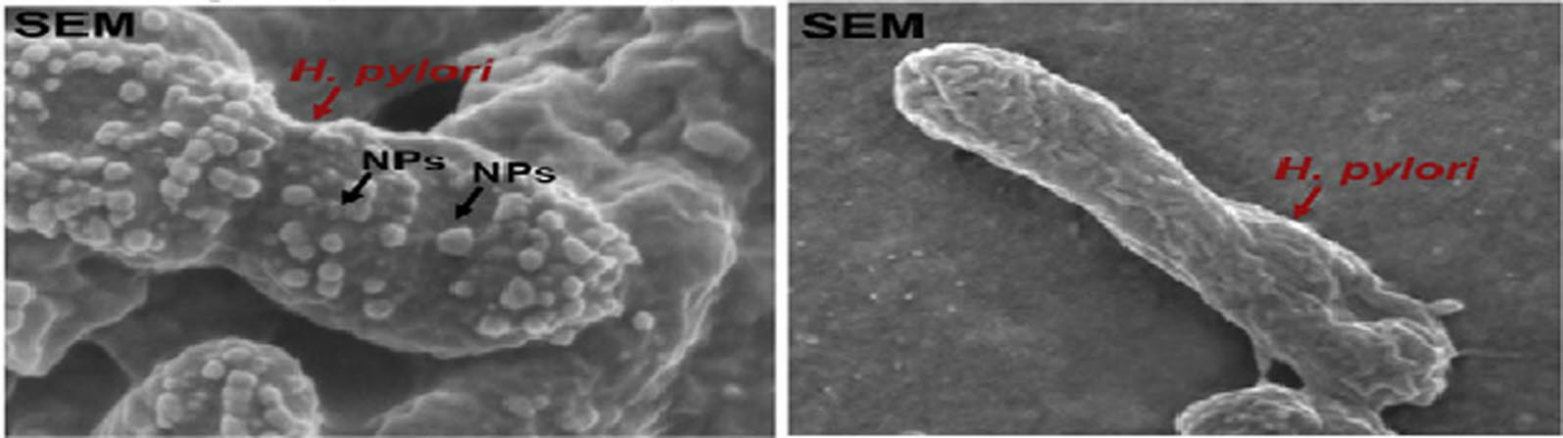
SEM micrograph of *H. pylori*: Left—*H. pylori* with fucose coated (AMX) NP and right—*H. pylori* control. Dots on bacteria surface—fucose coated (AMX) NP. Adapted with permission from [52]

Fucose-chitosan/heparin NP crosslinked with genipin and loaded with AMX, were also developed to target *H. pylori* lectins [52]. When tested in vitro*,* these NP successfully bound to *H. pylori* 26695 strain (Fig. 7). In addition, fucose coated (AMX) NP showed the highest growth inhibition (54%), followed by uncoated (AMX) NP (39%) and soluble AMX (24%). In vivo studies using C57BL/6J mice showed that fucose coated (AMX) NP achieved higher reduction of *H. pylori* load and induced less gastric cell inflammation than free AMX [52], supporting the potential of these nanoparticles.

In a subsequent study, a nanosystem containing fucose-chitosan/gelatin was used in combination with epigallocatechin-3-gallate (EGCG), a bioactive compound usually found in green tea that has antibacterial and anti-urease activity against *H. pylori* [60, 61]. The need of EGCG encapsulation was related with its instability in the stomach and incapacity to reach *H. pylori* at the target site (surface of epithelial gastric cells) [62]. In vitro assays using *H. pylori* 26695 strain showed that EGCG loaded NP had higher antimicrobial activity than EGCG in solution. However, these NP had low antimicrobial efficiency (≈30% growth inhibition). In vivo*,* fucose–chitosan/gelatin/EGCG NP also inhibited *H. pylori* growth, decreasing the bacteria load when compared with mice treated only with EGCG in solution [62]. Although this strategy is promising, there is still space for improvement, since only a 40–50% reduction in the bacterial load was achieved in vivo [62]. This work inspired the development of nanocomposites containing carboxymethyl chitosan (CMC) coupled with gold (Au) NP with antimicrobial properties using fucose and EGCG [33]. However, the new nanocomposites (fucose-CMC/EGCG/Au nanocomposites) did not present a significant improvement from the strategy previously described (in vitro antimicrobial effect against *H. pylori* ≈30% at the highest concentration) [33]. Another strategy used fucose-conjugated chitosan (C) NP loaded with berberine (BE), a natural compound with known antibacterial and anti-inflammatory properties [63]. The loaded BE fucose-C NP inhibited *H. pylori* 26695 strain growth in a dose dependent manner, reaching 37% at the maximum BE concentration (more 12% of growth inhibition than BE in solution). After infection of AGS cells with *H. pylori*, these nanoparticles were added to determine if a colocalization of labeled *H. pylori* and NP would be possible to observe by fluorescence microscopy on bacteria already adhered to cells. This colocalization was confirmed, demonstrating that BE fucose-C NP were able to reach bacteria adhered to cells. In C57BL/6 mice, BE fucose-C NP decreased the bacterial load in approximately 50% versus the 33% obtained using BE. However, no controls without fucose were done to determine its influence in NP specificity and activity [63].

More recently, fucoidan, a polysaccharide composed by fucose that is commonly found in algae [64], was used as NP coating to target *H. pylori* (planktonic and biofilms) [65]. The NP were constituted by: (i) metformin to improve host cell lysosomal activity [66]; (ii) linoleic acid (LA), a polyunsaturated fatty acid with anti-*H. pylori* activity [67, 68] and (iii) ebselen (EB), an urease inhibitor [69]. The interaction between fucoidan and *H. pylori* SS1 strain biofilm was demonstrated by fluorescence microscopy, with fucoidan coated NP showing better biofilm penetration and antimicrobial performance than the non-coated NP. Additionally, fucoidan coated NP decreased 90% of the *H. pylori* biofilm biomass [65]*.* This anti-biofilm performance was boosted by the fucoidan interference with *H. pylori* adhesion to gastric epithelial cells. Also, the combination of the blockage of urease activity by EB and the antibacterial effect of LA allowed the elimination of dispersed bacteria from the disintegrated biofilms [65]. Moreover, it was demonstrated that EB and LA reduced the oxidative stress both in vitro and in vivo*,* diminishing gastric epithelial cells exposure to reactive oxygen species (ROS) that promote cellular damage and trigger carcinogenesis during *H. pylori* infection [65]. While common triple therapy decreased the bacterial load but did not achieve eradication, these NP were able to reach 60% of *H. pylori* eradication rate in C57BL/6 mice [65], establishing a promising approach to eradicate *H. pylori* without using antibiotics.

Another strategy resourced to the use of mannose conjugated into chitosan NP (Man-C NP), which was tested against *H. pylori* antibiotic resistant clinical isolates [54]. Man-C inhibitory effect on *H. pylori* lectin was confirmed by molecular simulations, confirming the ability of this strategy to efficiently target *H. pylori* [54]. Moreover, it was observed by scanning and transmission electron microscopy (SEM/TEM) that the interaction between Man-C NP and *H. pylori* lead to a pronounced disruption of the bacteria membrane when compared with C NP [54]. Both Man-C NP and C NP were effective against *H. pylori* with Man-C NP achieving a slightly higher (5.7 log CFU/mL) bacterial load reduction than C NP (5.3 log CFU/mL) after 24 h. Similarly, when the NP were evaluated against *H. pylori* biofilms, Man-C NP promoted a higher reduction of biofilm thickness (75%) than C NP (55%) [54].

#### Phosphatidylethanolamine

The phospholipid phosphatidylethanolamine (PE) is one of the major components of eukaryotic and prokaryotic cellular membranes [70]. The PE present in *H. pylori* membrane acts as a steroid-binding lipid aiding the assimilation of free cholesterol, a crucial event for the bacterium survival, acquisition of resistance to antibiotics and evasion of host immune system [71]. However, *H. pylori* also has a PE binding protein in its membrane that recognizes PE in host cells, promoting the adhesion to the antrum of the human stomach [72]. Initially it was thought that this interaction was done through adhesins, but it was later discovered that the binding was promoted by *H. pylori* catalase expressed at the surface of bacterial membrane (also involved in the uptake of cholesterol) [72–75].

Taking advantage of the fact that PE can be used to form liposomes (LP), “dual function” LP were developed: PE targeted *H. pylori* catalase, while fucose was aimed to bind and block the bacterium BabA adhesin. In this strategy different liposomal formulations were prepared using cholesterol conjugated with fucose and epikuron 170 as a source of PE. To attain antimicrobial effect, ampicillin and metronidazole (MTZ) were encapsulated into all LP. As controls, LP without PE were prepared by switching epikuron 170 by 1,2-dipalmitoyl-sn-glycero-3-phosphocholine (DPPC) [76]. The interaction between LP labeled with 2-(12-(7-nitrobenz-2-oxa-1,3-diazol-4-yl)amino)dodecanoyl-1-hexadecanoyl-*sn*-glycero-3-phosphocholine (NBD-PC) and *H. pylori* 17875 (BabA^+^) and 149C (BabA^−^) strains was observed by epifluorescence microscopy using different LP formulations: fucose-PE-LP, PE-LP, fucose-DPPC-LP and DPPC-LP.

PE-LP (with and without fucose) interacted scarcely with both *H. pylori* 149C (BabA^−^) and *H. pylori* 17875 (BabA^+^). Opposite wise, DDPC-PL (with and without fucose) interacted more with both strains since a higher fluorescent intensity was observed [76]. In fact, fucose-DDPC-LP interacted more with *H. pylori* 17875 (BabA^+^) than fucose-PE-LP, which may indicate that PE is not influencing the *H. pylori* targeting. However, since PE-LP (with and without fucose) were negatively charged and DDPC-LP (with and without fucose) were neutral, this could be promoting electrostatic repulsion between the NP and the anionic bacterial membrane. Still, these results are merely qualitative (comparison of fluorescence intensity) and, despite being stated that preliminary antimicrobial assays were done using these LP loaded with ampicillin and that an antimicrobial effect was observed, these results were not disclosed [76].

Singh et al*.* designed double liposomes (DL; smaller liposomes inside lipid bilayers) using PE, phosphatidylcholine, cholesterol and stearylamine. Additionally, ranitidine bismuth citrate (RBC) and AMX were added to the formulations (AMX-RBC-DL) as antimicrobial agents [77]. The DL were tested in vitro against *H. pylori* 26695 strain to assess its ability to inhibit bacterial growth. AMX-RBC-DL achieved 87% of inhibition, being 3 times more effective than free AMX (27%) and the combination of AMX-RBC (73%) [77]. An agglutination assay demonstrated that *H. pylori* only agglutinated in the presence of DL, proving their binding to the bacteria. However, assays using DL without PE were not done as control to determine the specificity of PE binding [77]. For both cases (LP and DL), the targeting activity may not be exclusively related to the presence of PE, since cholesterol was used in the formulations. As *H. pylori* constantly uptakes this steroid to incorporate it in its membrane, cholesterol presence can also impact the targeting potential of these strategies.

In another study, nanolipobeads, spherical bipartite structures made of a hydrogel core enclosed within a lipid bilayer [78], were designed using a PE bilayer and a poly vinyl alcohol nanoparticles (PVA NP) core, also incorporating AMX and RBC onto the nanolipobeads for bactericidal performance [79]. When tested against *H. pylori* SKP56 strain, the nanolipobeads had higher antibacterial activity, achieving 80% of growth inhibition, whereas the control (free AMX-RBC) only reached 49%. Moreover, in situ adherence assays using gastric tissue sacs showed that *H. pylori* adhesion to gastric tissue was hindered when bacteria were preincubated with nanolipobeads. Altogether, these results established that the PE in nanolipobeads binds to their specific surface receptors in *H. pylori,* inhibiting adhesion to cells and improving the delivery of AMX and RBC in the bacterium vicinity. Complete eradication (100%) was observed using infected albino rats treated with nanolipobeads, while only a 33% eradication rate was observed for the control (AMX-RBC). However, nanolipobeads without PE were not tested as control to compare their specificity [79].

**Fig. 8:**
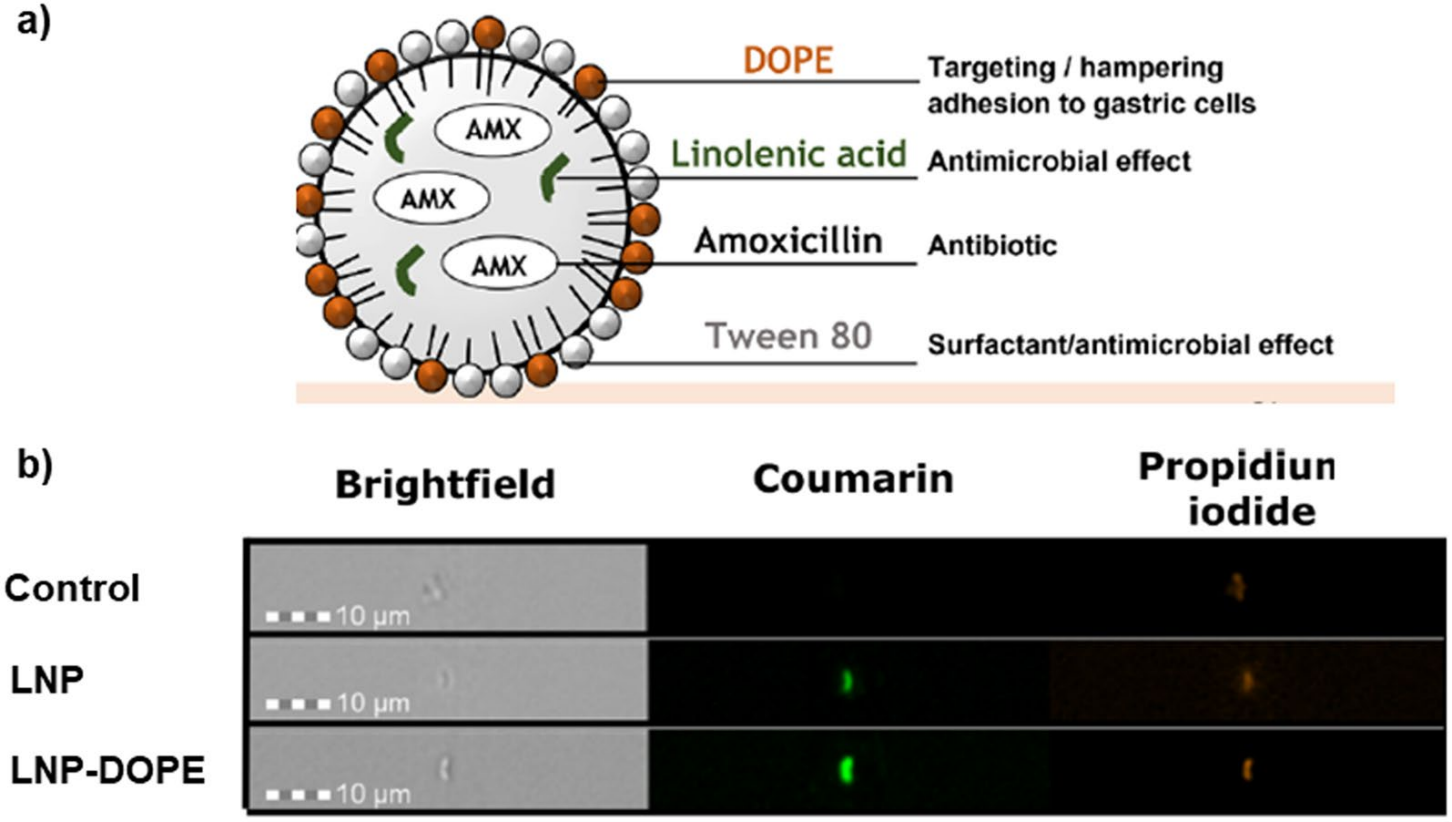
**a** Schematic representation of AMX-loaded LNP composition and **b** Imaging flow cytometry images of the interaction between coumarin-6-labeled LNP (green) and *H. pylori* J99 strain labeled with propidium iodide (orange). Control: *H. pylori* not exposed to LNP. Magnification: 40x. Adapted with permission from [80]

More recently, lipid nanoparticles (LNP) were produced using dioleoylphosphatidylethanolamine (DOPE) as source of PE and with AMX and linolenic acid to promote antimicrobial activity. The effects of each LNP component against *H. pylori* are described in Fig. 8a. As a control, LNP without DOPE was used [80].

The ability of PE to target *H. pylori* J99 strain was assessed by imaging flow cytometry. After *H. pylori* J99 strain incubation with fluorescent LNP for 15 min, 97% of bacteria were labeled with DOPE-LNP, while only 82% of *H. pylori* were labeled with LNP without DOPE (Fig. 8b). Importantly and confirming the anti-cell adhesion effect of PE strategy, it was seen that *H. pylori* adhesion to gastric cells (MKN-74 cell line) substantially decreased in the presence of DOPE-LNP (33%) versus the 70% obtained when using LNP without DOPE. Both formulations achieved complete *H. pylori* eradication in vitro and by SEM it was established that both disrupted *H. pylori* membrane. When tested in an in vitro infection model (2D cell culture using Transwell^®^ inserts), DOPE-LNP had the higher antimicrobial effect in *H. pylori* previously attached to MKN-74 cells (> 1 log CFU/mL reduction) [80]. DOPE-LNP showed an overall better performance than LNP without DOPE, confirming the targeting potential of PE for an in situ delivery of antimicrobial compounds. However, when comparing the results from both in vitro assays, DOPE-LNP activity was lower in the 2D model than when tested directly against the bacteria.

#### Epithelial cell membranes

**Fig. 9:**
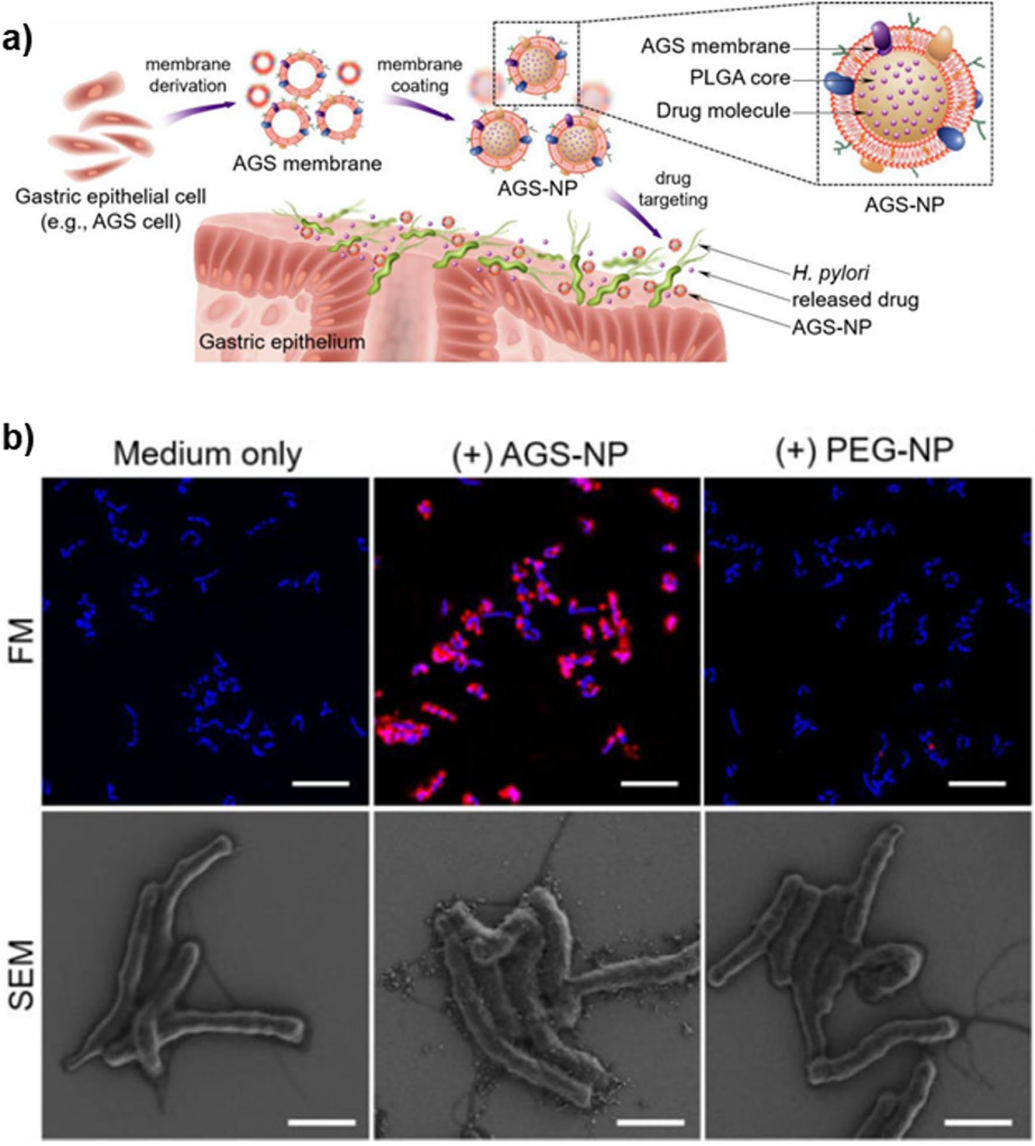
**a** Schematic illustration of the preparation of gastric epithelial cell (e.g. AGS cell) membrane-coated nanoparticles (AGS-NP) and their use for targeted antibiotic delivery to treat *H. pylori* infection and **b** Fluorescence microscopy (FM) and scanning electron microscopy (SEM) images of *H. pylori* (labeled with DAPI, blue) after incubation with medium only and NP with or without AGS (labeled with DiD, red). FM scale bar—5 μm and SEM scale bar—1 μm. Adapted with permission from [81]

To compete for bacterial adhesion to gastric cells, polylactic-co-glycolic acid (PLGA) NP were coated with plasma membranes derived from gastric epithelial cells, namely AGS cells (Fig. 9a). To improve the treatment outcome, AGS NP were also loaded with CLR (CLR-AGS NP) [81]. Eradication of *H. pylori* SS1 strain was only observed for CLR-AGS NP at the highest CLR concentrations (4–8 µg/mL) in opposite to CLR in solution and CLR-PLGA NP that did not have bactericidal effect at the same concentrations [81]. Additionally, it was observed by fluorescence microscopy and SEM that only CLR-AGS NP were co-localized with the bacterium, confirming the targeting properties of the AGS membrane coating (Fig. 9b). When tested in infected C57BL/6 mice, CLR-AGS NP had better bactericidal performance than the controls (CLR in solution and CLR-NP), decreasing the bacterial burden in more than 3 log CFU/g of stomach tissue. However, the in vivo eradication rate was set at 25%, probably because CLR-AGS NP activity can be hampered by competition with gastric cell epithelium [81].

Overall, all the above-described strategies successfully targeted *H. pylori* and reduced its ability to adhere to gastric cells. By hampering this important step in the establishment of *H. pylori* infection, these strategies can turn the bacterium more susceptible to the in situ delivered antimicrobial agents.

### Bind *H. pylori* UreI channel

**Fig. 10:**
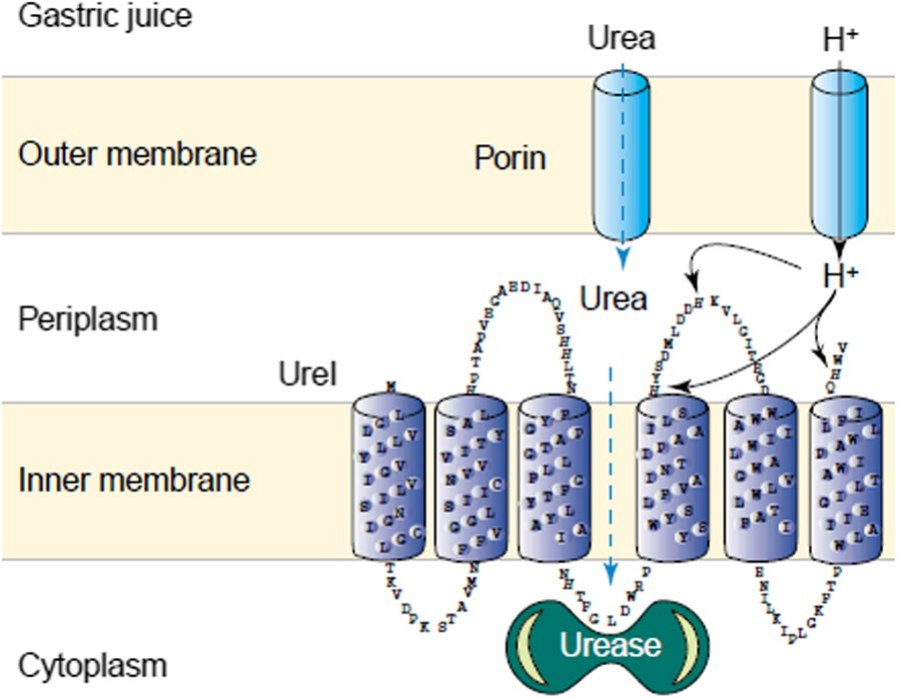
UreI channel is active between pH 2 and 6.5, allowing the entry of urea and consequently promoting the activity of urease. Adapted with permission from [87]

To survive in the gastric acidic environment *H. pylori* produces urease, an enzyme that converts endogenous urea in ammonia and carbon dioxide, increasing the pH at its vicinity [82, 83]. The transport of urea across *H. pylori* membrane is mediated by the urea channel UreI (Fig. 10) [84]. UreI is a pH-regulated channel that opens in low pH, promoting the uptake of urea and facilitating *H. pylori* urease activity. This active urea transport progressively decreases or stops when the pH reaches neutral levels [85, 86].

Aiming to target *H. pylori* UreI channel and to enable the delivery of antimicrobial compounds, urea-based nanosystems were developed [88, 89].

Qaiser et al*.* designed complex multitask nanomicelles containing: (i) urea (Ur) for UreI targeting; (ii) hyaluronic acid (mucoadhesive polymer) to increase their retention time in the stomach [90, 91]; (iii) papain (PAP; mucolytic enzyme) to improve their penetration through the mucus layer [92]; (iv) oleic acid (OA; antimicrobial fatty acid) and (v) CLR for improving antimicrobial effect (CLR-PAP-Ur-OA-nanomicelles) [88]. Efficacy assays showed a growth inhibition of *H. pylori* clinical isolates close to 100% after 48 h of incubation with CLR-PAP-Ur-OA-nanomicelles, whereas less growth inhibition was observed when CLR-OA-nanomicelles without Ur and PAP (70%) and CLR in solution (≈40%) were used [88]. When tested in *H. pylori* infected Wistar rats the results were similar, with targeted CLR-PAP-Ur-OA-nanomicelles having the highest reduction on the bacterial load, namely a decrease in CFUs between 7 and 30-fold (dose-dependent) when compared with untreated rats [88]. Nanomicelles without urea and PAP were less effective in vitro and in vivo, highlighting the importance of urea for *H. pylori* targeting and PAP for mucus penetration [88].

Another *H. pylori* UreI channel targeted nanomicelles were developed using carboxymethyl chitosan (CMCS) [93]. In this strategy, Cong et al*.* grafted ureido-groups (U) onto CMCS previously conjugated with stearic acid (SA). These nanomicelles loaded with CLR (CLR-U-CMCS-SA) [93] were bacteriostatic against *H. pylori* in a CLR concentration dependent way. At the highest CLR concentration, nanomicelles without ureido-groups were approximately 6 times less effective, supporting their targeting potential [93], which was further confirmed since only fluorescently labeled nanomicelles containing urea (CLR-U-CMCS-SA) were observed surrounding the bacteria. In vivo retention studies demonstrated that, after 24 h, the CLR-U-CMCS-SA nanomicelles were still present in the mice stomach, proving its effective retention on the target site and their potential to be used for prolonged drug release.

**Fig. 11:**
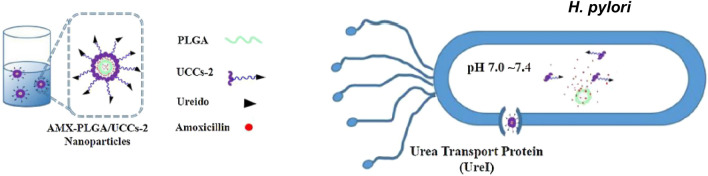
Schematic representation of AMX-PLGA/UCCs-2 NP composition and specific interaction with *H. pylori* UreI channel. Not to scale. Adapted with permission from [30]

Other studies reported the development of UreI-targeted nanoparticles (NP) by grafting urea onto chitosan before NP production. For that, chitosan was reacted with ureidododecanoic acid to produce two types of ureido-conjugated chitosan (UCCs-1 and UCCs-2) that were then used for NP production [89]. To improve treatment, NP were loaded with AMX. When tested in vitro both blank UCCs NP (without AMX) were ineffective against *H. pylori* 26695 strain. However, after 6 h of incubation, AMX UCCs NP yielded a faster bacteriostatic effect inducing 50% of growth inhibition versus the 27% obtained with the control (AMX chitosan NP). If incubated with *H. pylori* for longer periods (24 h), UCCs-2 NP achieved 87% of growth inhibition, while UCCs-1 NP were similar to the control without urea (80% and 78%, respectively) [89]. To evaluate the efficacy of these NP in physiologic conditions, their antimicrobial activity and specific binding was tested in the presence of urea. After 6 h, the growth inhibition of both UCCs-NP decreased with the increase of urea concentration. Moreover, UCCs-2 NP specific binding to *H. pylori* was demonstrated by flow cytometry using fluorescent UCCs-2 NP: when urea in solution increased, the uptake of UCCs-2 NP by *H. pylori* was reduced [89]. However, these nanoparticles were unstable at acidic pH, revealing an uncontrolled AMX release pattern that can compromise its clinical application. As such, new AMX-UCCs-2 NP using polylactic-co-glycolic acid (PLGA) (Fig. 11) with low drug release in pH 1.2 (gastric fluid) and higher release in pH 6–7 (gastric mucosa/epithelium) were formulated [30].

PLGA, a commonly used polymer in controlled drug delivery systems, can easily form core–shell nanoparticles with chitosan and its derivatives via electrostatic interactions [94]. AMX-PLGA/UCCs-2 NP were more active against *H. pylori* 26695 strain (20–60% growth inhibition) than the same formulation without the ureido conjugate UCCs-2 (15–40% growth inhibition). When tested in vivo, a higher reduction of *H. pylori* burden ( < 1 log CFU) was observed when *H. pylori* infected BALB/c mice were treated with AMX-PLGA/UCCs-2 NP in comparison with those treated with NP without UCCs-2 ( < 0.5 log CFU) [30].

**Fig. 12:**
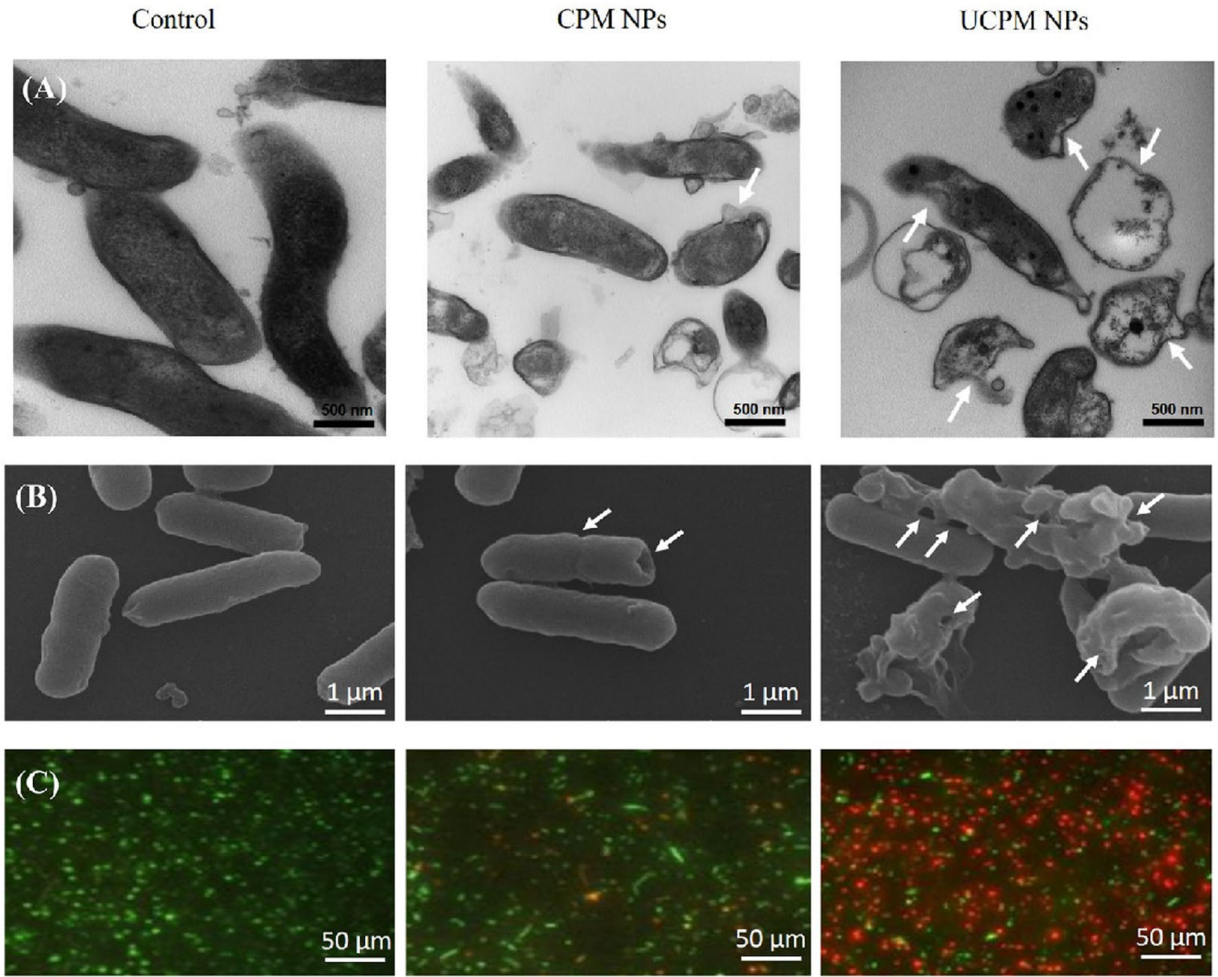
*H. pylori* treated with or without CPM-NPs and UCPM-NPs. **A** Transmission electron microscopy (TEM) images. Scale: 500 nm; **B** Scanning electron microscopy (SEM) images. The white arrows denote transmembrane pores formed by UCPM-NPs. Scale: 1 µm. **C** Fluorescence micrographs. Green—live bacteria; Red—dead bacteria. Scale: 50 µm. Used with permission from [95]

Another strategy developed by Arif et al*.* resourced to ureido-chitosan NP combined with carbon dots (CDs) to improve their antimicrobial potential [95]. CDs are stable, biocompatible and generally non-cytotoxic NP that have the potential to disrupt bacteria membranes by causing oxidative stress [96]. Thus, ureido-chitosan/poly(malic acid) NP were conjugated with CDs (UCPM NP) aiming to disrupt the bacterial membrane by ROS production and to enhance the transport of molecules through the bacterial membrane. TEM and SEM images showed that UCPM NP and NP without urea (CPM NP) promoted membrane and cytoplasmatic damage (Fig. 12A and B). Furthermore, UCPM NP had higher antimicrobial activity than CPM NP, confirming the specificity of urea NP to *H. pylori* (Fig. 12C) [95]. Additionally, mucus penetration assays were conducted in a 2D model (Transwell^®^) and it was observed that UCPM NP crossed the mucin layer and effectively killed *H. pylori*. To improve their antimicrobial efficacy, AMX was loaded into the UCPM NP. AMX-UCPM NP were effective against *H. pylori* 26695 strain in an AMX concentration dependent way, reaching *H. pylori* eradication at an AMX concentration of 0.75 µg/mL. Moreover, histological analysis of gastric tissue of C57BL/6 mice that were treated with AMX-UCPM NP showed lower *H. pylori* load than the non-treated control. Also, AMX-UCPM NP prevented alterations in the gastric mucosa in opposite to untreated mice, where cellular damages from infection (ulcers) were observed [95]. Although no quantification of the final *H. pylori* load was done, the targeting potential of UCPM NP was successfully demonstrated.

The above-mentioned strategies proved the efficacy of UreI channel-targeted drug delivery systems to improve antibiotic delivery inside *H. pylori*. However, the presence of urea secreted by the host gastric epithelium may compete with these approaches and compromise its effectiveness. Furthermore, since UreI channel closes at neutral pH, these strategies may not be able to target *H. pylori* adhered to the gastric epithelium, which could hinder effective eradication [85, 86]. However, they have the potential to be used as preventive strategies against *H. pylori* by acting upon bacteria on the mucus layer that is not yet in the gastric epithelium surroundings.

### *H. pylori* binding

#### *H. pylori* specific binding using antibodies

Antibodies (Ab) are proteins produced and recruited by the immune system to identify and neutralize foreign agents, like bacteria and viruses. Ab have great affinity and specificity towards an intended target and can be classified according to the number of epitopes that they are able to identify and bind: monoclonal if only a single epitope is recognized or polyclonal if several epitopes are recognized [97].

The use of a monoclonal Ab against *H. pylori* (*Hp* Ab) conjugated to liposomes (*Hp* Ab-LP) was explored by Wang et al*.* in 2022 to specifically target and kill *H. pylori* using sonodynamic therapy (SDT), a therapeutic strategy based on ultrasound that generates ROS and lead to bacteria/cell death [98, 99]. For that, the commonly used sonosensitizer indocyanine green (ICG) was incorporated into the above-mentioned liposomes (*Hp* Ab-LP-ICG) [98]. *Hp* Ab-LP-ICG, as well as the controls (free ICG and LP-ICG without *Hp* Ab), were incubated with the bacterium and ICG intrinsic fluorescence was used to test the specificity of the formulations. Labeled *H. pylori* was only detected when incubated with *Hp* Ab-LP-ICG, proving their effective targeting action in vitro. Furthermore, the use of ultrasounds induced bacterial lysis in an ICG concentration dependent way. Lastly, after treatment with *Hp* Ab-LP-ICG *H. pylori* was not detected in infected BALB/c mice (using a 13C-Urea breath test) [98].

**Fig. 13:**
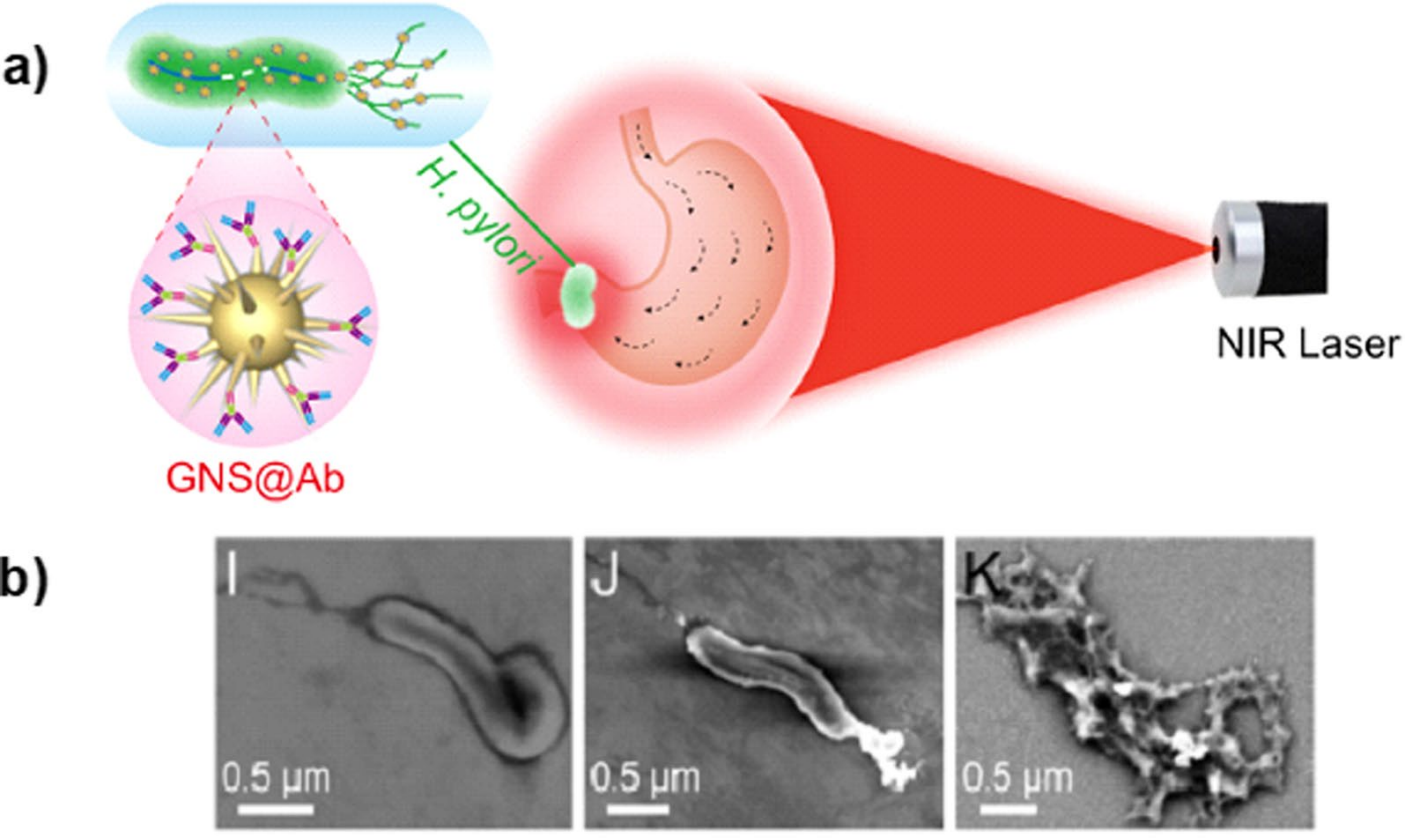
**a** Schematic representation of GNS-Ab application for targeted imaging and photothermal therapy. **b** SEM images of GNS-Ab targeting *H. pylori*. I—control *H. pylori* without GNS-Ab, J- GNS-Ab targeting *H. pylori* without photothermal treatment and K—GNS-Ab targeting* H. pylori* with photothermal treatment. Adapted with permission from [100]

Another approach used a modified *H. pylori* polyclonal Ab conjugated with gold nanostars (GNS) [100]. This conjugation intended to achieve a photodynamic therapy (PDT) application, that uses light to stimulate the generation of ROS consequently killing the bacteria. In this strategy the authors explored the GNS potential to produce ROS when exposed to near-infrared (NIR) laser irradiation [101–103]. No interaction was observed between GNS without Ab and *H. pylori* or GNS-Ab with *Escherichia coli*, demonstrating the Ab selectivity towards *H. pylori*. GNS-Ab eradicated 40 *H. pylori* clinical isolated strains with antibiotic resistance profile after the application of NIR laser irradiation (Fig. 13). When tested in BALB/c mice*,* GNS-Ab also eradicated *H. pylori* without affecting the gut microbiota [100].

These Ab based strategies have great potential for the development of specific SDT and PDT against *H. pylori*. Additionally, both systems have the potential to be used in a theragnostic approach, since they can be followed in real time by photoacoustic or ultrasound imaging techniques [104, 105].

#### *H. pylori* binding using non-specific targets

Other nano/microsystems were designed to eradicate *H. pylori* by targeting the bacterial membrane, their extracellular polymeric substances (EPS) or by using high temperature requirement A (HtrA) inhibitors. Although not specific towards *H. pylori* since these targets are present in other bacteria species, these strategies were specifically designed to be applied within gastric settings, having mucoadhesive or pH responsive properties that make them suitable for the quest against *H. pylori*. A brief overview of these systems will be given on the next subsections.

##### Bacterial membrane targeting

Dextran sulfate (DS) is a biocompatible polysaccharide commonly used in the medical field to mimic heparan sulfate [106, 107]. Several bacteria, including *H. pylori*, can adhere to heparan sulfate located in epithelial cells via heparan-binding proteins present in its membrane [108, 109]. To evaluate its potential as *H. pylori* targeting, DS was used as a coating for lysozyme nanoemulsions (NE) loaded with curcumin, a phytocompound with known antimicrobial activity against *H. pylori*. When tested against *H. pylori* J99 strain in an agar diffusion test, the DS-NE had a larger inhibition zone than the uncoated NE. Additionally, it was observed by flow cytometry a decrease in bacterial adhesion to AGS cells when *H. pylori* was pre-treated with DS-NE, confirming the anti-adhesion potential of this strategy [110].

**Fig. 14:**
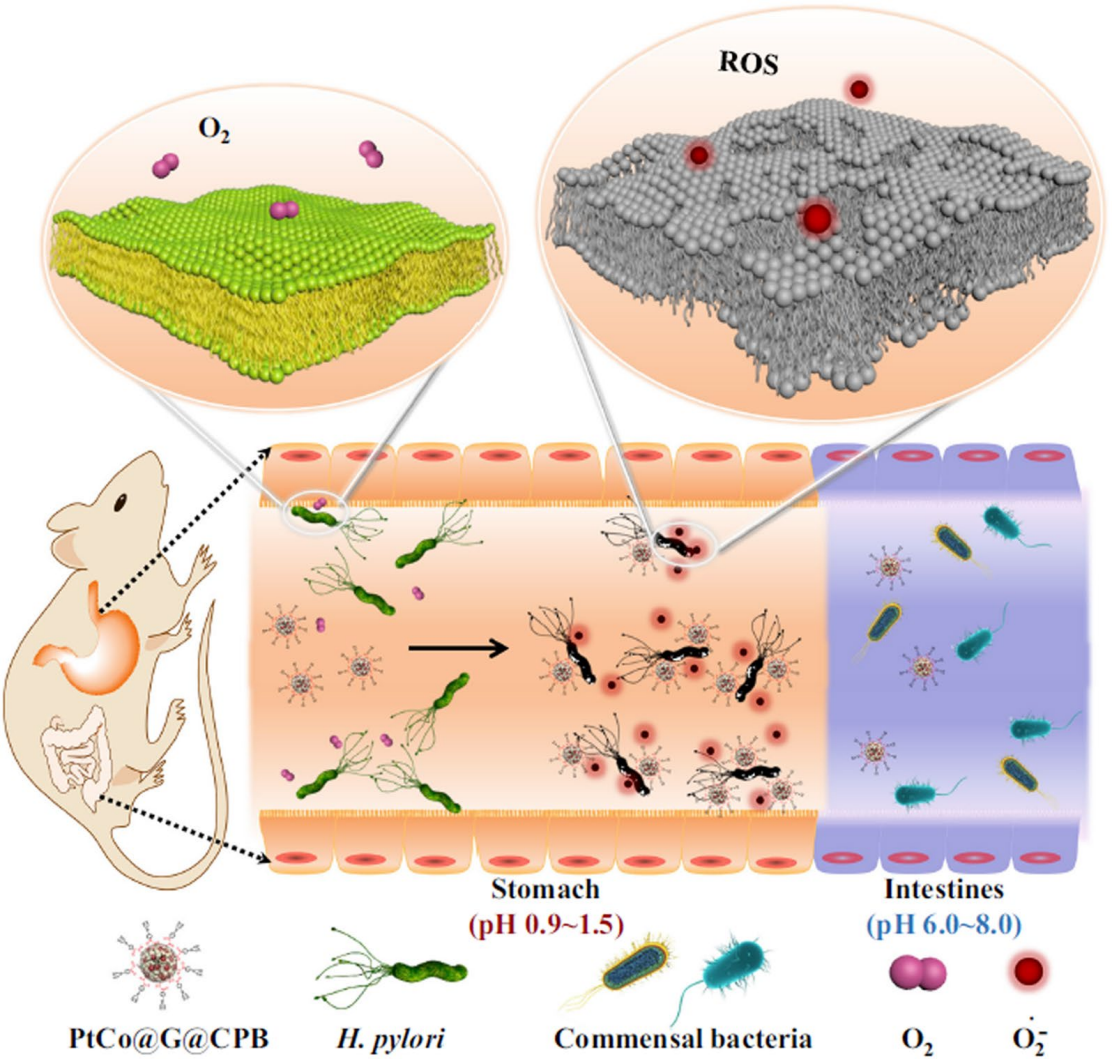
Schematic representation of the effect of nanozymes (PtCo-G-CPB) in vivo against *H. pylori*. The acidic pH promotes the specific targeting of *H. pylori* and the formation of ROS on stomach, not affecting the intestinal commensal bacteria. Used with permission from [112]

Other strategy designed for *H. pylori* binding using nonspecific targets was based on the production of nanozymes using boronic acid, an organic compound that binds to bacterial peptidoglycan [111–113]. Nanozymes are nanomaterials with enzyme-like characteristics that can exhibit antimicrobial activity using different mechanisms: (i) production of ROS, aiming to disrupt the bacterial membrane and to promote DNA or protein damage or (ii) DNase-like activity to damage extracellular DNA, whose integrity is important for bacterial interactions and biofilm formation [114]. Two different nanozymes for *H. pylori* treatment were developed, one composed by graphene-isolated platinum cobalt (PtCo-G) nanocrystals coated with C_18_-PEG_n_-benzeneboronic acid (CPB) [112] and other with a persistent luminescence NP (PLNP) core coated with mesoporous silica (MS) and gold (Au) NP functionalized with chitosan-benzeneboronic acid [113]. These nanozymes were pH-responsive, since their oxidase- and peroxidase-like activity promotes the formation of ROS, predominantly under acidic pH [115, 116] (Fig. 14) and both were bactericidal against *H. pylori *in vitro. In vivo*,* these nanozymes were only active in acidic gastric pH, not affecting the intestinal commensal bacteria [112, 113]. Regarding *H. pylori* effect, PtCo-G-CPB nanozymes were bactericidal when tested in BALB/c mice, achieving similar effect to the control triple therapy (OMZ, AMX, and CLR). MS-PLNP-Au-chitosan-benzeneboronic acid nanozymes also attained a decrease in bacterial load (qualitative analysis).

##### Extracellular polymeric substances (EPS) targeting

Like several bacteria*, H. pylori* secretes extracellular polymeric substances (EPS), mostly exopolysaccharides that play an important role in bacterial growth and as biofilms component [117, 118]. Clay NP were designed against *H. pylori* EPS [119] having in its composition montmorillonite (M; a clay mineral that can attach onto EPS and exhibits mucoadhesive properties) tethered with a cationic linear polyethyleneimine (lPEI, disrupts bacterial membrane) and loaded with metronidazole (MTZ). MTZ-M-IPEI NP successfully eradicated *H. pylori *in vitro in a MTZ concentration dependent way. Free MTZ and IPEI-MTZ without montmorillonite showed a lower effect, suggesting that the higher activity of MTZ-M-IPEI NP is related to *H. pylori* targeting promoted by the clay. MTZ-M-IPEI NP decreased *H. pylori* load in infected BALB/c mice, as observed by histology studies (no quantitative analysis was done) [119].

##### High temperature requirement A (HtrA) inhibitors

HtrA protease is ubiquitously expressed in bacteria, being essential for their survival in unfavorable conditions. *H. pylori* HtrA is secreted extracellularly helping bacteria survival in the harsh gastric environment [120]. Thus, HtrA inhibitors have the potential to shut down *H. pylori* mechanisms of protection, promoting bacterial death. Some nano/micro systems were developed using known HtrA inhibitors, namely JO146 and apigenin [121, 122]. Different size PLGA-JO146 NP were produced and tested against *H. pylori* ATCC^®^ 43504 strain. The minimum bactericidal concentration (MBC) of PLGA-JO146 NP was reached at lower concentration (12.5 µM) than free JO146 (25 µM). Regarding PLGA NP, the MBC was not achieved at the concentrations tested [122]. Also, a microsponge was produced using the polymer Eudragit RS 100 and apigenin. When tested against *H. pylori* ATCC^®^ 43504 strain, both apigenin alone or incorporated in the microsponge inhibited *H. pylori* growth, proving the targeting and antimicrobial properties of apigenin. Moreover, although the microsponge required twice the apigenin concentration to achieve the MIC (16 µg/mL), it prolonged its effect for more 36 h compared to free apigenin [121].

The specific characteristics of the nano/micro approaches herein described, namely the binding molecules and particle composition, antibiotics encapsulated, level of experimentation and efficacy rates, are summarized in Table 2.

## Conclusions

Overall, promising results were obtained when bioengineering was used in the development of targeted nanotherapeutics for *H. pylori* infection. Nano/microparticles (NP/MP) were designed to block *H. pylori* adhesion to gastric cells, namely using coatings that are specifically recognized by *H. pylori* OMP (e.g. glycans) or that bind to *H. pylori* glycoproteins (e.g. fucose, mannose). Additionally, urea-based strategies involving the UreI channel also had successful targeting results. Although in most cases encapsulation of antimicrobial drugs, namely antibiotics, was needed to achieve *H. pylori* eradication, these NP/MP were advantageous when compared with non-targeted NP/MP and free drugs, either because they blocked bacteria adhesion to gastric cells and/or allowed the delivery of drugs in situ or even inside the bacterium. However, the efficiency of these strategies that mimic compounds expressed in host gastric cells (e.g. Leb antigens, cell membranes, urea) as coatings may be affected by competitive binding, which can hamper their efficacy in vivo. Also, a direct comparison between the different approaches herein reported is difficult since they are in different development stages, with some only showing in vitro data while other strategies have already completed pre-clinical (in vivo*)* testing. Nevertheless, the most promising strategies were the ones using antibodies for a highly specific *H. pylori* binding coupled with SDT and PDT therapies, and the use of pH-responsive nanozymes that bound to *H. pylori* membrane, killing bacteria by the production of ROS. All these antibiotic-free formulations excelled in targeting *H. pylori*, coupled with good performance in vitro and in vivo without affecting the gut microbiota. Moreover, due to their photoacoustic, photoluminescence or magnetic imaging properties they can be further explored as theragnostic tools. Nonetheless, it is important to strain that some strategies that underwent in vivo testing were only evaluated qualitatively or fell below the 90% eradication rate recommended by the Maastricht Consensus [14]. Altogether, there is still room for improvement. Also, so far, none of these strategies reached clinical trials, which may be linked with scale-up problems, or “simply” to the “long road” of enrolling in a clinical trial. Nevertheless, with the failing rates of the conventional therapy available to counteract *H. pylori*, it is imperative to translate these promising *H. pylori* targeting systems from bench to the clinics.

## Data Availability

Not applicable.

## Abbreviations

Ab: Antibodies
AHA: Acetohydroxamic acid
AMX: Amoxicillin
Au: Gold
BabA: Blood group antigen-binding adhesin
CDs: Carbon dots
CFU: Colony forming units
ChMP: Chitosan microspheres
CLR: Clarithromycin
CMCS: Carboxymethyl chitosan
ConA: Concanavalin A
C: Chitosan
DAPI: 4′,6-Diamidino-2-phenylindole
DiD: 1,1-Dioctadecyl-3,3,3,3- tetramethylindodicarbocyanin
DL: Double liposomes
DOPE: Dioleoylphosphatidylethanolamine
DPPC: 1,2-Dipalmitoyl-sn-glycero-3-phosphocholine
DS: Dextran sulfate
e.g.: Exempli gratia
EB: Ebselen
EGCG: Epigallocatechin-3-gallate
EPS: Extracellular polymeric substances
FDA: Food and drug administration
FITC: Fluorescein isothiocyanate
FU: Fucose
G: Gliadin
GNS: Gold nanostars
H*.**pylori*: *Helicobacter* *pylori*
HtrA: High temperature requirement A
ICG: Indocyanine green
LA: Linoleic acid
Le^b^: Lewis b
LLA: Linolenic acid
LNP: Lipid nanoparticles
LPN: Lipid polymer nanocarrier
Man: Mannose
MP: Microparticles
MS: Mesoporous silica
MTZ: Metronidazole
NE: Nanoemulsions
ND: No data
NP: Nanoparticles
OMZ: Omeprazole
OA: Oleic acid
OMP: Outer membrane proteins
PAP: Papain
PDT: Photodynamic therapy
PE: Phosphatidylethanolamine
PECS: Pectin sulfate
PLGA: Polylactic-co-glycolic acid
PVA: Polyvinyl alcohol
RBC: Ranitidine bismuth citrate
ROS: Reactive oxygen species
SA: Stearic acid
SabA: Sialic acid-binding adhesin
SDT: Sonodynamic therapy
SEM: Scanning electron microscopy
sLe^x^: Sialyl-Lewis x
TEM: Transmission electron microscopy
UCCs: Ureido-conjugated chitosan
UEA-I: *Ulex europaeus* Agglutinin I
WHO: World Health Organization
